# Correlates of sunscreen use among high school students: a cross-sectional survey

**DOI:** 10.1186/1471-2458-11-679

**Published:** 2011-08-31

**Authors:** Carolyn J Heckman, Elliot J Coups

**Affiliations:** 1 Cancer Prevention and Control Program, Fox Chase Cancer Center, 333 Cottman Avenue, Philadelphia, PA 19111, USA; 2 The Cancer Institute of New Jersey, 195 Little Albany Street, New Brunswick, NJ 08901, USA; 3 Department of Medicine, UMDNJ-Robert Wood Johnson Medical School, 125 Paterson Street, New Brunswick, NJ 08901, USA; 4 Department of Health Education and Behavioral Science, UMDNJ-School of Public Health, 683 Hoes Lane West, Piscataway, NJ 08854, USA

**Keywords:** sunscreen, adolescents, Integrative Model, skin cancer prevention, intentions

## Abstract

**Background:**

Adolescents put themselves at risk of later skin cancer development and accelerated photo-aging due to their high rates of ultraviolet radiation exposure and low rates of skin protection. The purpose of the current study was to determine which of the Integrative Model constructs are most closely associated with sunscreen use among high school students.

**Methods:**

The current study of 242 high school students involved a survey based on the Integrative Model including demographic and individual difference factors, skin protection-related beliefs and outcome evaluations, normative beliefs, self-efficacy, sunscreen cues and availability, intentions, and sunscreen use. Our analyses included multiple linear regressions and bootstrapping to test for mediation effects.

**Results:**

Sunscreen use was significantly associated with female gender, greater skin sensitivity, higher perceived sunscreen benefits, higher skin protection importance, more favorable sunscreen user prototype, stronger skin protection norms, greater perceived skin protection behavioral control, and higher sunscreen self-efficacy. Intentions to use sunscreen mediated the relationships between most skin protection-related beliefs and sunscreen use.

**Conclusions:**

The current study identified specific variables that can be targeted in interventions designed to increase sunscreen use among adolescents.

## Background

Skin cancer is the most common form of cancer, with over a million new cases diagnosed annually in the United States [1]. The prevalence of melanoma, the deadliest form of skin cancer, has been increasing over the past 30 years [2] and is now the second most common cancer among women in their twenties [3]. However, skin cancers are largely preventable with engagement in recommended protective practices, such as limiting ultraviolet radiation (UV) exposure, wearing sun-protective clothing, and using sunscreen. Long-term use of sunscreen is associated with decreased risk of non-melanoma skin cancers [4]. Results of research studies examining the association between sunscreen use and melanoma risk have been mixed [4,5]. However, these studies have typically been limited by a number of methodological issues, including retrospective reports of sunscreen use and the use of non-randomized designs. The results of a recent prospective randomized controlled trial of sunscreen use found a lower incidence of invasive melanoma among individuals assigned to a sunscreen intervention compared to those in the control condition [6]. Promoting routine sunscreen use as a component of skin protection is a critical aspect of public health approaches designed to reduce the incidence of skin cancer [7].

Skin protection is especially important for children and adolescents. Early intense exposure to UV radiation is associated with higher rates of skin cancer [8-12], and regular sunscreen use during childhood and adolescence could reduce lifetime incidence of non-melanoma skin cancers by approximately 78% [13]. Adolescence, in particular, is a critical period for skin cancer prevention because adolescents and young adults have the lowest skin protection rates of all age groups [14], receive large amounts of UV radiation [15-17], and increase their UV exposure habits as they move into adulthood and are less influenced by their parents [10,18]. In 2003, only 14% of US high school students reported routine sunscreen use [19]. In some cases, higher risk adolescents are less likely to protect their skin. For example, White Hispanic high school students in Miami, Florida were twice as likely to never or rarely wear sunscreen as non-White Hispanics [20]. While several interventions have been found to produce short-term increases in sunscreen use among children, their long-term effect among adolescents is questionable [18]. Thus, it is important to better understand the factors underlying adolescents' use of sunscreen and other skin protection behaviors so that we can intervene more effectively with those at highest-risk of developing skin cancer.

Fishbein's Integrative Model (IM; [21]) provides a comprehensive theoretical framework to describe the relationships among variables predicting adolescents' skin protection intentions and behavior. Drawing from several empirically-validated health behavior theories, the IM includes multiple categories of predictor variables, including: background/individual difference variables, beliefs, norms, self-efficacy, intentions, contextual factors, and behavior. The beliefs category includes behavioral outcome beliefs, defined as beliefs about the consequences of performing the behavior (i.e., what will happen if I apply sunscreen), and outcome evaluations, defined as subjective evaluations or favorability of these consequences. Norms can include prototypes (evaluation of the typical person who engages in the behavior) and subjective norms (the extent to which associates engage in the behavior), as well as motivation to comply with these norms. Self-efficacy includes perceived control over the behavior and self-efficacy to perform the behavior. Contextual factors include environmental cues to engaging in the behavior. These beliefs, norms, self-efficacy, and cues contribute to behavioral intentions, which in turn influence behavior.

Prior research studies have identified associations between adolescent skin protection including sunscreen use and several variables drawn from the IM; however, no prior study has evaluated the full IM within the same study and sample. In terms of *background and individual difference variables*, factors that have been found to be associated with greater sunscreen use among adolescents include white race, female gender, younger age, higher skin sensitivity, greater knowledge of sun protection recommendations, and a family history of skin cancer [22-25]. *Behavioral beliefs *associated with adolescent sunscreen use include a preference for natural/light skin, greater perceived benefits of sun protection, believing that it is not worth burning to get a tan, and perceiving shorter sun exposures as "safer" than longer ones [24]. *Normative factors *linked with adolescents' use of sunscreen include sunscreen information and modeling by friends, parental information provision and insistence on sunscreen use, and receipt of sun protection advice from health care providers [24]. *Self-efficacy *for skin protection is one of the variables that has been found to be most strongly associated with engagement in skin cancer protection, including among adolescents and young adults [26-29]. Perceived behavioral control over skin protection has also been found to be associated with skin protection [30]. No prior study has examined the association between sunscreen use and sunscreen-related *cues or availability*. The only IM construct that we did not include in this study was skills, since we did not expect there to be much variability in perceived skill level for sunscreen application. Among adolescents and young adults, skin protection *intentions *are associated with skin protection *behaviors *including sunscreen use [31-35].

The purpose of the current study was to determine which of the IM constructs are most closely associated with sunscreen use among adolescent high school students. Based on prior research, we expected that background/individual differences, beliefs, norms, and self-efficacy would all contribute to adolescent sunscreen use. However, in the current study, we included several novel variables within these domains that have not been investigated previously among high school students. These variables included beliefs about general health (i.e., health consciousness), sunscreen user prototype, as well as body image self-efficacy and emotional coping self-efficacy. Additionally, although not previously included in sun protection research, we expected cues and availability to be related to sunscreen use. Finally, we expected that intentions to use sunscreen would mediate the relationships between the IM variables and sunscreen use.

## Methods

### Procedures and Participants

Participants were recruited from health and science classes at a high school in Philadelphia. Students were invited to complete a twenty-minute paper and pencil survey after listening to a 30-minute lecture in the school auditorium on skin cancer and its prevention given by the first author. The lecture was given to 453 students during three class periods on the same day in April, 2010. The study was approved by the Fox Chase Cancer Center Institutional Review Board, and parents provided passive consent. A small number of students decided not to participate in the lecture, primarily because they had a recent family history of skin cancer and were concerned that they might be upset by the talk. After the lecture, the first author explained the study to the students and teachers and answered any questions. Students who were present for the lecture took the surveys with them and were asked to return them to their teachers within one week. Incentives were not provided, and no identifying information was requested on the surveys. A total of 253 questionnaires were returned with signed assent forms (22 surveys were returned without signed assent forms and were excluded from all analyses). Questionnaires from four participants showed evidence of systematic responding and were excluded from all analyses. Data regarding the sunscreen outcome measure were missing for an additional seven individuals, leaving an available sample size of *N *= 242.

### Measures

Unless otherwise stated, for each of the multi-item scales described below, we created a scale score by averaging item responses.

#### Background and individual difference variables

Participants reported their race/ethnicity, sex, and grade in school. Skin sensitivity was assessed using a single-item adapted version of the six-level Fitzpatrick [36] standard classification system (where I = burn easily/don't tan and VI = never burn/I am dark-skinned), with a lower score denoting skin that is more sensitive to the sun and prone to burning. Knowledge of skin cancer and skin cancer prevention was assessed using six items (e.g., "Some medications can make skin more sensitive to burning") adapted from previous research [37,38]. We summed the number of correct responses to the knowledge items. Participants indicated the number of people they know well who had been diagnosed with skin cancer.

#### Behavioral beliefs and outcome evaluations

Sunscreen benefits was assessed using five items (with a response scale from 1 = *strongly disagree *to 5 = *strongly agree*) (e.g., "Sunscreen is more trouble than it's worth) adapted from an existing measure [39]. The items were reverse-coded and averaged (α = .61), so that a high score represented a high level of sunscreen benefits. A single item with a five-point response scale assessed the importance (from 1 = *not at all *to 5 = *very*) of protecting the skin from ultraviolet damage [40,41]. Two items assessed perceived risk of getting skin cancer and looking old prematurely compared to other people of the same age (α = .77) [42]. Both items used a five-point response scale (from 1 = *much less *to 5 = *much more*). The perceived severity of skin cancer and looking old prematurely were assessed with four items that used a five-point response scale (from 1 = *strongly disagree *to 5 = *strongly disagree*) (α = .79) (adapted from Aiken et al. [43]). Participants' level of appearance orientation was measured using an 11-item subscale (α = .84) from the Multidimensional Body Self Relation Questionnaire [44]. Reponses were provided on a five-point scale (from 1 = *strongly disagree *to 5 = strongly disagree). Health consciousness was assessed with a 5-item subscale (α = .93) using a five-point response scale (from 1 = *not at all *to 5 = *very*) from the Health Orientation Scale [45].

#### Normative beliefs

Tanning norms were assessed using nine items drawn from previous research [33,46]. Two items had low item-total correlations and a scale score was calculated from the remaining seven items (e.g., "Most of my friends feel that a tan is a good thing") (α = .71). Four items drawn from previous research by Hillhouse and colleagues [46] assessed skin protection norms. Two of the items asked about perceptions of friends and family members about the participant's engagement in skin protection (using a response scale from 1 = *strongly think I should not *to 7 = *strongly think I should*). Two corresponding items asked the participant how motivated he/she was to comply with their friends' and family members' opinions (from 1 = *not at all motivated *to 7 = *completely motivated*). A total skin protection norms score was created by summing the products of the two friends' items and the two family members' items. Sunscreen user prototype was assessed by asking participants to indicate the extent to which each of six adjectives (from 1 = *not at all *to 7 = *very much*) describe the typical person who uses sunscreen frequently (adapted from Gibbons et al., [47]). The two reverse-coded adjectives (careless, self-centered) had low item-total correlations and thus a scale score was created by averaging across the remaining four favorable adjectives (e.g., smart, attractive) (α = .68).

#### Self-efficacy

Three items drawn from previous research [48] assessed participants' level of sunscreen self-efficacy (α = .80). Responses were given on a five-point scale (from 1 = *not at all *to 5 = *very*). We also assessed two other types of self-efficacy that we thought could potentially be related to skin protection. For example, the primary motivation for tanning is for appearance enhancement. Thus, the nine-item Body Image Self-Efficacy Scale [49] was used to measure confidence in the ability to maintain a positive body image in various situations (α = .90). The response scale was the same as that for the sunscreen self-efficacy items. We assessed emotional coping self-efficacy in the current study due to recent reports that frequent tanning may be related to emotional problems and tanning dependence [50-55]. Four items from the Coping Self-Efficacy Scale [56] assessed emotional coping self-efficacy, defined as individuals' confidence in their ability to stop unpleasant emotions and thoughts. A six-point response scale was used (from 1 = *not at all *to 6 = *extremely*) and responses were averaged across the items (α = .87). Perceived behavioral control was assessed using a single item (and a response scale from 1 = *very difficult *to 7 = *very easy*) that asked about the perceived ease of engaging in ultraviolet skin protection [46].

#### Sunscreen cues and availability

Participants who indicated that they had ever worn sunscreen (*n *= 232) were asked whether each of seven factors related to sunscreen cues or availability contributed to their wearing sunscreen on the last occasion they used it. The items asked about expectations of parents/guardians, personal rules and plans, borrowing sunscreen from others, and the convenience and costs of purchasing sunscreen. A yes/no response was used for each item.

#### Sunscreen intentions

Three items adapted from previous research [57] were used to assess sunscreen intentions. The items used a seven-point response scale (from 1 = *strongly disagree to *7 = *strongly agree*) (α = .83).

#### Use of sunscreen

Three items asked about use of sunscreen (from 1 = *never *to 5 = *always*) during the summer months when outside in the sun for more than 15 minutes (α = .92) [58].

### Statistical Analyses

Separately for each category of potential correlates of sunscreen use (i.e., background and individual difference variables, behavioral beliefs and outcome evaluations, normative beliefs, self-efficacy, sunscreen cues and availability), we conducted a multiple linear regression analysis with all of the correlates in the category as independent variables and use of sunscreen as the dependent variable. Again, for each category of correlates, we conducted analyses to test whether statistically significant associations between correlates and sunscreen use were mediated by sunscreen intentions. To test for mediation, we used a bootstrapping approach (using a macro developed by Preacher and Hayes [59]) with 5000 bootstrapped samples to determine the point estimate and bias-corrected and accelerated 95% confidence interval of each indirect effect [60,61]. Indirect effects for which the 95% confidence interval did not contain zero were denoted as showing statistically significant mediation. When testing for mediation, all correlates in a category were included as covariates in the model, and separate analyses were used to test each indirect effect [59]. Analyses were conducted using SPSS Statistics 17.0 and SAS 9.2, and a cutoff of *p *< .05 was used to determine statistical significance.

## Results

The characteristics of the study sample are shown in Table 1. The participants were 59% female and 86% non-Hispanic white. Almost half of the students reported knowing at least one person who had been diagnosed with skin cancer. Knowledge of skin cancer and its prevention was moderately high, with an average score of 4.36 correct out of the six items. The mean score on the intentions to use sunscreen measure was 3.46 (on a 1 to 7 scale) and the standard deviation was 1.45. For the measure of sunscreen use (which used a 1 to 5 scale), the mean and standard deviation were 2.86 and 1.04, respectively. The correlation between sunscreen intentions and use of sunscreen was *r *= .49 (*p *< .001). The results of the regression analyses examining correlates of sunscreen use are shown in Table 2 and summarized in the following sections.

**Table 1:** Characteristics of Study Sample (*N *= 242)

Variable	Sample %
Sex	
Male	40.9
Female	59.1
High school grade	
9^th^	38.8
10^th^	5.0
11^th^	26.0
12^th^	30.2
Race/ethnicity	
Hispanic	5.4
Non-Hispanic white	86.4
Non-Hispanic Asian	3.7
Non-Hispanic black	1.2
Non-Hispanic other	3.3
Fitzpatrick skin type	
I	11.6
II	12.5
III	18.3
IV	34.9
V	17.8
VI	5.0
Number of people know with skin cancer	
0	52.5
1	43.8
2	3.3
3	0.4
Knowledge of skin cancer and its prevention (*M *= 4.36, *SD *= 1.47)	
0	2.9
1	2.1
2	5.8
3	13.6
4	21.5
5	29.8
6	24.4

*Note. M *= mean; *SD *= standard deviation.

**Table 2:** Multiple Linear Regression Analyses and Bootstrapping Tests of Indirect Effects of Correlates of Sunscreen Use

Variables	Model *R*^2^	*b *(95% CI)	*p *Value	Point Estimate (95% CI) for Indirect Effect Mediated by Sunscreen Intentions
Demographics and Individual Differences	.17			
Sex^a^		-0.38 (-0.64, -0.12)	.004	-0.14 (-0.28, -0.02)
High school grade		-0.05 (-0.15, 0.04)	.291	
Race^b^		0.03 (-0.36, 0.42)	.891	
Fitzpatrick skin type		-0.25 (-0.35, -0.16)	< .001	-0.08 (-0.14, -0.03)
Know someone diagnosed with skin cancer		-0.10 (-0.36, 0.16)	.429	
Knowledge of skin cancer and its prevention		0.06 (-0.03, 0.14)	.187	
Behavioral Beliefs and Outcome Evaluations	.34			
Sunscreen benefits		0.58 (0.41, 0.74)	< .001	0.13 (0.06, 0.23)
Importance of protecting skin		0.28 (0.18, 0.39)	< .001	0.07 (0.03, 0.14)
Perceived risk of skin cancer/premature aging		0.11 (-0.01, 0.23)	.069	
Perceived severity of skin cancer/premature aging		0.05 (-0.10, 0.20)	.523	
Appearance orientation		0.10 (-0.07, 0.26)	.264	
Health consciousness		-0.03 (-0.16, 0.10)	.640	
Normative Beliefs	.12			
Sunscreen user prototype		0.15 (0.04, 0.27)	.008	0.04 (-0.01, 0.10)
Tanning norms		-0.01 (-0.19, 0.18)	.954	
Skin protection norms		0.01 (0.01, 0.02)	< .001	0.005 (0.002, 0.009)
Self-Efficacy	.30			
Perceived behavioral control		0.09 (0.02, 0.17)	.019	0.00 (-0.02, 0.02)
Sunscreen self-efficacy		0.50 (0.37, 0.62)	< .001	0.17 (0.08, 0.29)
Body image self-efficacy		-0.15 (-0.29, 0.00)	.044	-0.01 (-0.06, 0.03)
Emotional coping self-efficacy		0.02 (-0.02, 0.05)	.247	
Sunscreen Cues and Availability^c^	.28			
Expected to wear it by parents/guardians		0.41 (0.07, 0.75)	.018	0.15 (0.02, 0.33)
Always bring some		0.73 (0.44, 1.01)	< .001	0.19 (0.07, 0.35)
Purposely brought some		0.20 (-0.08, 0.47)	.161	
Happened to have some		-0.18 (-0.42, 0.07)	.165	
Borrowed some from someone else		0.08 (-0.17, 0.32)	.535	
Bought some because it was convenient to buy		0.33 (-0.02, 0.69)	.068	
Bought some because it was inexpensive		0.06 (-0.28, 0.40)	.737	

*Note. b *= unstandardized regression coefficient; CI = confidence interval. ^a ^Sex was coded as female = 1, male = 2. ^b ^Race was coded as minority = 1, non-minority = 2. ^c ^Each variable was coded as no = 0, yes = 1.

### Correlates of Sunscreen Use: Demographic and Individual Difference Variables

Among the demographic and individual difference variables, greater use of sunscreen was reported by female students and those with more sensitive skin. As shown in the final column of Table 2, each of these associations was mediated by sunscreen intentions, such that female students and those with more sensitive skin had higher sunscreen intentions, which in turn were positively associated with sunscreen use. Sunscreen use was not associated with high school grade, race, knowing someone with skin cancer, or knowledge of skin cancer and its prevention.

### Correlates of Sunscreen Use: Behavioral Beliefs and Outcome Evaluations

Individuals with stronger perceptions of sunscreen benefits or importance of protecting their skin were more likely to use sunscreen. Each of these associations was mediated by sunscreen intentions, with higher intentions among those reporting greater perceived sunscreen benefits or importance of skin protection. Sunscreen use was not associated with the perceived risk or severity of skin cancer and premature aging, appearance orientation, or health consciousness.

### Correlates of Sunscreen Use: Normative Beliefs

Students who had stronger skin protection norms or a more positive sunscreen user prototype reported greater sunscreen use. The association between skin protection norms and sunscreen use was mediated by sunscreen intentions, such that intentions were higher among those with stronger skin protection norms. Tanning norms were not associated with sunscreen use.

### Correlates of Sunscreen Use: Self-Efficacy

Students reported greater sunscreen use if they had a higher level of perceived behavioral control over skin protection or sunscreen self-efficacy, or a lower level of body image self-efficacy. The association between sunscreen self-efficacy and sunscreen use was mediated by sunscreen intentions, with higher intentions among those with greater sunscreen self-efficacy. Emotional coping self-efficacy was not associated with sunscreen use.

### Correlates of Sunscreen Use: Sunscreen Cues and Availability

Of the sunscreen cues and availability variables examined, students reported greater sunscreen use if they indicated that they were expected to wear it by their parents/guardians or if they always bring some with them when planning to be out in the sun. Each of these associations was mediated by sunscreen intentions, such that intentions were higher among individuals endorsing each item. Sunscreen use was not associated with the remaining sunscreen cues and availability variables examined.

## Discussion

The current study consisted of a survey of sunscreen use and its potential correlates among high school students. Consistent with the IM and prior research, sunscreen use was associated with female gender, greater perceived skin sensitivity, higher perceived sunscreen benefits, higher skin protection importance, stronger skin protection norms, greater perceived skin protection behavioral control, and higher sunscreen self-efficacy [23,24,26,28,62-65]. Prior studies have found sunscreen use to be associated with race and age, but not necessarily in consistent directions [22,23,66]. In the current study, there may not have been enough variability or a large enough sample of certain subgroups to identify such associations. We did not find sunscreen use to be associated with knowledge of skin cancer, knowing someone with skin cancer, perceived risk or severity of skin cancer and premature aging, appearance or health orientation, tanning norms, or emotional coping self-efficacy as it has been in some prior reports [23,25,66]. Overall, sunscreen use was more likely to be associated with positive attitudes and normative beliefs about sunscreen use and skin protection as opposed to negative attitudes toward skin cancer or photo-aging risks. One prior study found that gain-framed messages had a greater impact on increasing sunscreen use among beachgoers than loss-framed messages [67].

A novel finding of the study is the association of adolescent sunscreen use with a more favorable sunscreen user prototype. A prototype refers to an "image" norm (i.e., appeal of sunscreen users) as opposed to a "statistical" norm (i.e., how many of my friends wear sunscreen). Further exploration of perceived positive and negative characteristics of typical sunscreen users could be informative. Additionally, future research should explore whether role modeling and portraying sunscreen users as appealing could enhance skin protection interventions.

Interestingly, sunscreen use was associated with a low level of body image self-efficacy. Adolescents and young adults experience considerable pressure to appear tan and attractive [62,68,69]. Due to these societal norms for tan skin, body image self-efficacy may be lower among individuals with fair skin who cannot tan effectively and who must use sunscreen or other skin protection more often than others in order to prevent sunburns. On the other hand, individuals who can tan may do so in order to improve their body image. Thus, they may wear sunscreen more frequently simply because they are more frequently exposed to the sun. In a study of patients with body dysmorphic disorder, twenty-five percent reported tanning to address their body image concerns [70]. Additionally, low perceived physical attractiveness was found to be 'associated with indoor tanning among Swedish adolescents [71]. Thus, a focus on body image may be an important component of skin protection interventions for adolescents. Emotional coping self-efficacy was not associated with sunscreen use in the current sample, suggesting that skin exposure and protection are separate constructs rather than two ends of a continuum.

In terms of contextual factors, we found that always carrying sunscreen and parental expectations for wearing sunscreen were associated with sunscreen use among students. However, most of the sunscreen contextual items were not significantly associated with sunscreen use. The items that were significantly associated with sunscreen use pertained to norms and habits as opposed to availability and convenience, which may be important distinctions for future intervention efforts.

We found variables from all of the IM [22] construct categories to be associated with sunscreen use. Consistent with some prior research [27,33,66], most of the statistically significant associations between the correlates and sunscreen use were mediated by intentions. However, the relationships between sunscreen use and sunscreen user prototype, skin protection behavioral control, and body image self-efficacy were not mediated by intentions. This suggests that these correlates may be more proximal determinants of sunscreen use or that their associations may be mediated by factors other than sunscreen intentions. For example, adolescents may emulate the behavior of an appealing prototypical sunscreen user because they perceive him/her to be smart, attractive, and so on, but this may not necessarily be mediated by sunscreen intentions per se. Future research, particularly longitudinal studies, is warranted to examine this issue.

Strengths of the current study include the use of the IM as a comprehensive conceptual framework and inclusion of several novel correlates not previously examined in the context of skin cancer prevention. Limitations include the use of a sample of students from a single high school, the cross-sectional design which limits our ability to make causal inferences from the mediational analyses, and self-report of sunscreen use. However, most skin cancer prevention studies use self-report measures, and several studies have demonstrated the internal and test-retest reliability and criterion and concurrent validity of self-report skin protection behavior compared to observation and objective measures, with no systematic bias identified among various populations [72-74]. Participants may have had some difficulty accurately recalling summer sunscreen use in April; however, this factor was consistent across participants. The lecture may have affected the extent to which students endorsed some of the questionnaire items due to social desirability or a change in beliefs about skin cancer or sunscreen use. However, all students who took the survey had been exposed to the lecture, and the lecture would not be expected to influence the relationship among the variables, which was the primary focus of the study.

## Conclusions

Overall, these findings support the IM in that variables from each category of the model were significantly associated with sunscreen use, and these relationships were mediated by intentions to use sunscreen in most cases. However, this study provides more information than prior research about which specific types of background variables, beliefs, norms, and self-efficacy are most closely associated with sunscreen use among adolescents. These data suggest that greater effort is needed to increase sunscreen use among adolescent boys and teens with high body image self-efficacy. While individuals with lower skin sensitivity are less at risk for skin cancer [75,76], they still possess some risk for the disease as well as other types of skin damage such as photo-aging. Our study results also suggest that future interventions to promote sunscreen use among adolescents should target multiple beliefs, including sunscreen benefits, skin protection importance, favorable sunscreen user prototype, skin protection norms including parental norms, skin protection behavioral control, and sunscreen self-efficacy. Interventions that target such beliefs among adolescents may help increase their skin protection and thus decrease their risk of developing skin cancer.

## List of abbreviation

UV: ultraviolet radiation.

## Competing interests

The authors declare that they have no competing interests.

## Authors' contributions

CJH was responsible for data acquisition. EJC was responsible for data analysis. Both authors made substantial contributions to conception and design as well as interpretation of data; were involved in drafting the manuscript and revising it critically for important intellectual content; and have given final approval of the version to be published.

## Pre-publication history

The pre-publication history for this paper can be accessed here:

http://www.biomedcentral.com/1471-2458/11/679/prepub

## References

[B1] Stern RS Prevalence of a history of skin cancer in 2007: results of an incidence-based model Arch Dermatol 2010 146 3 279 282 10.1001/archdermatol.2010.4 20231498

[B2] American Cancer Society How many people get melanoma? http://www.cancer.org/Cancer/SkinCancer-Melanoma/OverviewGuide/melanoma-skin-cancer-overview-key-statistics Accessed December 29, 2010

[B3] Ibrahim SF Brown MD Tanning and cutaneous malignancy Dermatol Surg 2008 34 4 460 474 10.1111/j.1524-4725.2007.34092.x 18248469

[B4] Kutting B Drexler H Evaluation of skin-protective means against acute and chronic effects of ultraviolet radiation from sunlight Curr Probl Dermatol 2007 34 87 97 17312359 10.1159/000099606

[B5] Antoniou C Kosmadaki MG Stratigos AJ Katsambas AD Sunscreens-what's important to know J Eur Acad Dermatol and Venereol 2008 22 9 1110 1118 10.1111/j.1468-3083.2007.02580.x 18482317

[B6] Green AC Williams GM Logan V Strutton GM Reduced melanoma after regular sunscreen use: randomized trial follow-up J Clin Oncol 2011 29 3 257 263 10.1200/JCO.2010.28.7078 21135266

[B7] Gimotty PA Glanz K Sunscreen and melanoma: what is the evidence? J Clin Oncol 2011 29 3 249 250 10.1200/JCO.2010.31.7529 21135278

[B8] Cust AE Armstrong BK Goumas C Jenkins MA Schmid H Hopper JL Kefford RF Giles GG Aitken JF Mann GJ Sunbed use during adolescence and early adulthood is associated with increased risk of early-onset melanoma Intl J Cancer 2011 128 2425 2435 10.1002/ijc.25576 PMC2993823 20669232

[B9] Kricker A Armstrong BK Goumas C Litchfield M Begg CB Hummer AJ Marrett LD Theis B Millikan RC Thomas N Ambient UV, personal sun exposure and risk of multiple primary melanomas Cancer Causes Control 2007 18 3 295 304 10.1007/s10552-006-0091-x 17206532 PMC4206211

[B10] MacNeal RJ Dinulos JG Update on sun protection and tanning in children Curr Opin Pediatr 2007 19 4 425 429 10.1097/MOP.0b013e3282294936 17630607

[B11] Solomon CC White E Kristal AR Vaughan T Melanoma and lifetime UV radiation Cancer Causes Control 2004 15 9 893 902 10.1007/s10552-004-1142-9 15577291

[B12] Tatalovich Z Wilson JP Mack T Yan Y Cockburn M The objective assessment of lifetime cumulative ultraviolet exposure for determining melanoma risk J Photochemist Photobiol B 2006 85 3 198 204 10.1016/j.jphotobiol.2006.08.002 16963272

[B13] Stern R Weinstein M SG B Risk reduction for nonmelanoma skin cancer with childhood sunscreen use Arch Dermatol 1986 122 5 537 545 10.1001/archderm.122.5.537 3707169

[B14] Stanton WR Janda M Baade PD Anderson P Primary prevention of skin cancer: a review of sun protection in Australia and internationally Health Promot Internation 2004 19 3 369 378 10.1093/heapro/dah310 15306621

[B15] Baron ED Kirkland EB Domingo DS Advances in photoprotection Dermatol Nurs 2008 20 4 265 272 18819220

[B16] Coups EJ Manne SL Heckman CJ Multiple skin cancer risk behaviors in the U.S. population Am J Prev Med 2008 34 2 87 93 10.1016/j.amepre.2007.09.032 18201637

[B17] Heckman CJ Coups EJ Manne SL Prevalence and correlates of indoor tanning among US adults J Am Acad Dermatol 2008 58 5 769 780 10.1016/j.jaad.2008.01.020 18328594 PMC2601681

[B18] Eide MJ Weinstock MA Public health challenges in sun protection Dermatol Clin 2006 24 1 119 124 10.1016/j.det.2005.08.007 16311174

[B19] Jones SE Saraiya M Sunscreen use among US high school students, 1999-2003 J School Health 2006 76 4 150 153 10.1111/j.1746-1561.2006.00085.x 16536855

[B20] Ma F Collado-Mesa F Hu S Kirsner RS Skin cancer awareness and sun protection behaviors in white Hispanic and white non-Hispanic high school students in Miami, Florida Arch Dermatol 2007 143 8 983 988 10.1001/archderm.143.8.983 17709656

[B21] Fishbein M Cappella JN The role of theory in developing effective health communications Journal of Communication 2006 56 S1 S17 10.1111/j.1460-2466.2006.00280.x

[B22] Cokkinides V Weinstock M Glanz K Albano J Ward E Thun M Trends in sunburns, sun protection practices, and attitudes toward sun exposure protection and tanning among US adolescents, 1998-2004 Pediatrics 2006 118 3 853 864 10.1542/peds.2005-3109 16950974

[B23] Geller AC Colditz G Oliveria S Emmons K Jorgensen C Aweh GN Frazier AL Use of sunscreen, sunburning rates, and tanning bed use among more than 10 000 US children and adolescents Pediatrics 2002 109 6 1009 1014 10.1542/peds.109.6.1009 12042536

[B24] Kasparian NA McLoone JK Meiser B Skin cancer-related prevention and screening behaviors: a review of the literature J Behav Med 2009 32 5 406 428 10.1007/s10865-009-9219-2 19521760

[B25] Keesling B Friedman HS Psychosocial factors in sunbathing and sunscreen use Health Psychol 1987 6 5 477 493 3678173 10.1037//0278-6133.6.5.477

[B26] Hay JL Oliveria SA Dusza SW Phelan DL Ostroff JS Halpern AC Psychosocial mediators of a nurse intervention to increase skin self-examination in patients at high risk for melanoma Cancer Epidemiol Biomarkers Prev 2006 15 6 1212 1216 10.1158/1055-9965.EPI-04-0822 16775183

[B27] Hillhouse JJ Adler CM Drinnon J Turrisi R Application of Azjen's theory of planned behavior to predict sunbathing, tanning salon use, and sunscreen use intentions and behaviors J Behav Med 1997 20 4 365 378 10.1023/A:1025517130513 9298435

[B28] James AS Tripp MK Parcel GS Sweeney A Gritz ER Psychosocial correlates of sun-protective practices of preschool staff toward their students Health Educ Res 2002 17 3 305 314 10.1093/her/17.3.305 12120846

[B29] Myers LB Horswill MS Social cognitive predictors of sun protection intention and behavior Behav Med 2006 32 2 57 63 10.3200/BMED.32.2.57-63 16903615

[B30] Branstrom R Ullen H Brandberg Y Attitudes, subjective norms and perception of behavioural control as predictors of sun-related behaviour in Swedish adults Prev Med 2004 39 5 992 999 10.1016/j.ypmed.2004.04.004 15475034

[B31] de Vries H Lezwijn J Hol M Honing C Skin cancer prevention: behaviour and motives of Dutch adolescents European J Cancer Prev 2005 14 1 39 50 10.1097/00008469-200502000-00006 15677894

[B32] Jackson KM Aiken LS A psychosocial model of sun protection and sunbathing in young women: the impact of health beliefs, attitudes, norms, and self-efficacy for sun protection Health Psychol 2000 19 5 469 478 11007155 10.1037//0278-6133.19.5.469

[B33] Jackson KM Aiken LS Evaluation of a multicomponent appearance-based sun-protective intervention for young women: uncovering the mechanisms of program efficacy Health Psychol 2006 25 1 34 46 16448296 10.1037/0278-6133.25.1.34

[B34] Mahler HI Kulik JA Butler HA Gerrard M Gibbons FX Social norms information enhances the efficacy of an appearance-based sun protection intervention Soc Sci Medi 2008 67 2 321 329 10.1016/j.socscimed.2008.03.037 PMC2491400 18448221

[B35] White KM Robinson NG Young RM Anderson PJ Hyde MK Greenbank S Rolfe T Keane J Vardon P Baskerville D Testing an extended theory of planned behaviour to predict young people's sun safety in a high risk area Br J Health Psychol 2008 13 Pt 3 435 448 17535506 10.1348/135910707X210004

[B36] Fitzpatrick TB The validity and practicality of sun-reactive skin types I through VI Arch Dermatol 1988 124 6 869 871 10.1001/archderm.124.6.869 3377516

[B37] Lazovich D Forster J Sorensen G Emmons K Stryker J Demierre MF Hickle A Remba N Characteristics associated with use or intention to use indoor tanning among adolescents Arch Pediatr Adolesc Med 2004 158 9 918 924 10.1001/archpedi.158.9.918 15351760

[B38] Stryker JE Lazovich D Forster JL Emmons KM Sorensen G Demierre MF Maternal/female caregiver influences on adolescent indoor tanning J Adolesc Health 2004 35 6 528. e521-529 15581535 10.1016/j.jadohealth.2004.02.014

[B39] Mahler HI Kulik JA Gibbons FX Gerrard M Harrell J Effects of appearance-based interventions on sun protection intentions and self-reported behaviors Health Psychol 2003 22 2 199 209 12683740 10.1037//0278-6133.22.2.199

[B40] Daeppen JB Bertholet N Gmel G Gaume J Communication during brief intervention, intention to change, and outcome Subst Abus 2007 28 3 43 51 10.1300/J465v28n03_05 18077302

[B41] Harris TR Walters ST Leahy MM Readiness to change among a group of heavy-drinking college students: correlates of readiness and a comparison of measures J Am Coll Health 2008 57 3 325 330 10.3200/JACH.57.3.325-330 18980889

[B42] Ford JS Ostroff JS Hay JL Buckley TR Stein TR Berwick M Primavera LH Shike M Participation in annual skin cancer screening among women seeking routine mammography Prev Med 2004 38 6 704 712 10.1016/j.ypmed.2004.01.017 15193890

[B43] Aiken LS West SG Woodward CK Reno RR Health beliefs and compliance with mammography-screening recommendations in asymptomatic women Health Psychol 1994 13 2 122 129 8020455 10.1037//0278-6133.13.2.122

[B44] Brown TA Cash TF Mikulka PJ Attitudinal body-image assessment: factor analysis of the Body-Self Relations Questionnaire J Pers Assess 1990 55 12 135 144 2231236 10.1080/00223891.1990.9674053

[B45] Snell WE Johnson G Lloyd PJ Hoover MW The Health Orientation Scale: A measure of psychological tendencies associated with health Eur J Personality 1991 5 169 183 10.1002/per.2410050208

[B46] Hillhouse JJ Turrisi R Kastner M Modeling tanning salon behavioral tendencies using appearance motivation, self-monitoring and the theory of planned behavior Health Educ Res 2000 15 4 405 414 10.1093/her/15.4.405 11066458

[B47] Gibbons FX Gerrard M Lane DJ Mahler HI Kulik JA Using UV photography to reduce use of tanning booths: a test of cognitive mediation Health Psychol 2005 24 4 358 363 16045371 10.1037/0278-6133.24.4.358

[B48] Weinstock MA Rossi JS Redding CA Maddock JE Randomized controlled community trial of the efficacy of a multicomponent stage-matched intervention to increase sun protection among beachgoers Prev Med 2002 35 6 584 592 10.1006/pmed.2002.1114 12460526

[B49] Pinto AM Guara AS Heinberg LJ DiClimente CC Development of the eating disorder recovery self-efficacy questionnaire Int J Eat Disord 2006 39 5 376 384 10.1002/eat.20256 16528731

[B50] Harrington CR Beswick TC Leitenberger J Minhajuddin A Jacobe HT Adinoff B Addictive-like behaviours to ultraviolet light among frequent indoor tanners Clin Exp Dermatol 2010 1 6 10.1111/j.1365-2230.2010.03882.x 20545951

[B51] Heckman CJ Egleston BL Wilson DB Ingersoll KS A preliminary investigation of the predictors of tanning dependence Am J Health Behav 2008 32 5 451 464 18241130 10.5555/ajhb.2008.32.5.451 PMC4089889

[B52] Hillhouse J Turrisi R Shields AL Patterns of indoor tanning use: implications for clinical interventions Arch Dermatol 2007 143 12 1530 1535 10.1001/archderm.143.12.1530 18087003

[B53] Mosher CE Danoff-Burg S Addiction to indoor tanning: relation to anxiety, depression, and substance use Arch Dermatol 2010 146 4 412 417 10.1001/archdermatol.2009.385 20404230 PMC3756887

[B54] Nolan BV Feldman SR Ultraviolet tanning addiction Dermatol Clin 2009 27 2 109 112 v 10.1016/j.det.2008.11.007 19254653

[B55] Nolan BV Taylor SL Liguori A Feldman SR Tanning as an addictive behavior: a literature review Photodermatol Photoimmunol Photomed 2009 25 1 12 19 10.1111/j.1600-0781.2009.00392.x 19152511

[B56] Chesney MA Neilands TB Chambers DB Taylor JM Folkman S A validity and reliability study of the coping self-efficacy scale Br J Health Psychol 2006 11 Pt 3 421 437 16870053 10.1348/135910705X53155 PMC1602207

[B57] Mahler HI Kulik JA Harrell J Correa A Gibbons FX Gerrard M Effects of UV photographs, photoaging information, and use of sunless tanning lotion on sun protection behaviors Arch Dermatol 2005 141 3 373 380 10.1001/archderm.141.3.373 15781679

[B58] Weinstock MA Rossi JS Redding CA Maddock JE Cottrill SD Sun protection behaviors and stages of change for the primary prevention of skin cancers among beachgoers in southeastern New England Ann Behav Med 2000 22 4 286 293 10.1007/BF02895664 11253439

[B59] Preacher KJ Hayes AF Asymptotic and resampling strategies for assessing and comparing indirect effects in multiple mediator models Behav Res Methods 2008 40 3 879 891 10.3758/BRM.40.3.879 18697684

[B60] Mackinnon A Griffiths KM Christensen H Comparative randomised trial of online cognitive-behavioural therapy and an information website for depression: 12-month outcomes Br J Psychiatry 2008 192 2 130 134 10.1192/bjp.bp.106.032078 18245031

[B61] Shrout PE Bolger N Mediation in experimental and nonexperimental studies: new procedures and recommendations Psychol Methods 2002 7 4 422 445 12530702

[B62] Adams MA Norman GJ Hovell MF Sallis JF Patrick K Reconceptualizing decisional balance in an adolescent sun protection intervention: Mediating effects and theoretical interpretations Health Psychol 2009 28 2 217 225 19290714 10.1037/a0012989 PMC2657937

[B63] Hillhouse JJ Stair AW Adler CM Predictors of sunbathing and sunscreen use in college undergraduates J Behav Med 1996 19 6 543 561 10.1007/BF01904903 8970914

[B64] Maddock JE Redding CA Rossi JS Weinstock MA Development and validation of an appearance motivation attitudes scale for sun protection Psychol Health 2005 20 6 775 788 10.1080/14768320500165944

[B65] Mosher CE Danoff-Burg S Social predictors of sunscreen and self-tanning product use J Am Coll Health 2005 54 3 166 168 10.3200/JACH.54.3.166-168 16335316

[B66] Mermelstein RJ Riesenberg LA Changing knowledge and attitudes about skin cancer risk factors in adolescents Health Psychol 1992 11 6 371 376 1286656 10.1037//0278-6133.11.6.371

[B67] Detweiler JB Bedell BT Salovey P Pronin E Rothman AJ Message framing and sunscreen use: gain-framed messages motivate beach-goers Health Psychol 1999 18 2 189 196 10194055 10.1037//0278-6133.18.2.189

[B68] Cafri G Thompson JK Roehrig M van den Berg P Jacobsen PB Stark S An investigation of appearance motives for tanning: The development and evaluation of the Physical Appearance Reasons For Tanning Scale (PARTS) and its relation to sunbathing and indoor tanning intentions Body Image 2006 3 3 199 209 10.1016/j.bodyim.2006.05.002 18089223

[B69] Manne S Lessin S Prevalence and correlates of sun protection and skin self-examination practices among cutaneous malignant melanoma survivors J Behav Med 2006 29 5 419 434 10.1007/s10865-006-9064-5 16855870

[B70] Phillips KA Conroy M Dufresne RG Menard W Didie ER Hunter-Yates J Fay C Pagano M Tanning in body dysmorphic disorder Psychiatry Q 2006 77 2 129 138 10.1007/s11126-006-9002-2 PMC1622896 16779685

[B71] Boldeman C Jansson B Nilsson B Ullen H Sunbed use in Swedish urban adolescents related to behavioral characteristics Prev Med 1997 26 1 114 119 10.1006/pmed.1996.9986 9010906

[B72] Glanz K Mayer JA Reducing ultraviolet radiation exposure to prevent skin cancer methodology and measurement Am J Prev Med 2005 29 2 131 142 10.1016/j.amepre.2005.04.007 16005810

[B73] Glanz K McCarty F Nehl EJ O'Riordan DL Gies P Bundy L Locke AE Hall DM Validity of self-reported sunscreen use by parents, children, and lifeguards Am J Prev Med 2009 36 1 63 69 10.1016/j.amepre.2008.09.012 18945582 PMC2626407

[B74] O'Riordan DL Glanz K Gies P Elliott T A pilot study of the validity of self-reported ultraviolet radiation exposure and sun protection practices among lifeguards, parents and children Photochem Photobiol 2008 84 3 774 778 10.1111/j.1751-1097.2007.00262.x 18179624 PMC3725580

[B75] Tadokoro T Kobayashi N Zmudzka BZ Ito S Wakamatsu K Yamaguchi Y Korossy KS Miller SA Beer JZ Hearing VJ UV-induced DNA damage and melanin content in human skin differing in racial/ethnic origin The FASEB Journal 2003 17 9 1177 1179 12692083 10.1096/fj.02-0865fje

[B76] Veierod MB Weiderpass E Thorn M Hansson J Lund E Armstrong B Adami HO A prospective study of pigmentation, sun exposure, and risk of cutaneous malignant melanoma in women J Natl Cancer Inst 2003 95 20 1530 1538 14559875 10.1093/jnci/djg075

